# T Cell Activation in South African HIV-Exposed Infants Correlates with Ochratoxin A Exposure

**DOI:** 10.3389/fimmu.2017.01857

**Published:** 2017-12-22

**Authors:** Lianna Frances Wood, Matthew P. Wood, Bridget S. Fisher, Heather B. Jaspan, Donald L. Sodora

**Affiliations:** ^1^Department of Microbiology and Immunology, Albert Einstein College of Medicine, New York, United States; ^2^Center for Infectious Disease Research, Seattle, WA, United States; ^3^Divisions of Paediatrics, Institute of Infectious Disease and Molecular Medicine, University of Cape Town, Cape Town, South Africa; ^4^Department of Infectious Disease, Seattle Children’s Research Institute, Seattle, WA, United States

**Keywords:** infant, feeding, breastfeeding, contamination, HIV

## Abstract

The introduction of non-breastmilk foods to HIV-infected infants is associated with increased levels of immune activation, which can impact the rate of HIV disease progression. This is particularly relevant in countries where mother-to-child transmission of HIV still occurs at unacceptable levels. The goal of this study was to evaluate the levels of the toxic food contaminant ochratoxin A (OTA) in HIV-exposed South African infants that are either breastfed or consuming non-breast milk foods. OTA is a common mycotoxin, found in grains and soil, which is toxic at high doses but has immunomodulatory properties at lower doses. Samples from HIV-exposed and HIV-unexposed infants enrolled in prospective observational cohort studies were collected and analyzed at birth through 14 weeks of age. We observed that infants consuming non-breast milk foods had significantly higher plasma levels of OTA at 6 weeks of age compared to breastfed infants, increasing until 8 weeks of age. The blood levels of OTA detected were comparable to levels observed in OTA-endemic communities. OTA plasma levels correlated with HIV target cell activation (CCR5 and HLADR expression on CD4+ T cells) and plasma levels of the inflammatory cytokine CXCL10. These findings provide evidence that elevated OTA levels in South African infants are associated with the consumption of non-breastmilk foods and activation of the immune system. Reducing infant OTA exposure has the potential to reduce immune activation and provide health benefits, particularly in those infants who are HIV-exposed or HIV-infected.

## Introduction

Exclusive breastfeeding is strongly recommended by the World Health Organization for all infants, irrespective of HIV status. Exclusive breastfeeding has been associated with reductions in rates of gastrointestinal infections, childhood asthma, obesity, and childhood leukemia (1). Furthermore, exclusively breastfed infants have been shown to have lower levels of pro-inflammatory bacterial taxa in their stool and reduced gut permeability when compared to formula-fed infants (2–4). In addition to providing unique nutritional and immunological benefits, exclusive breastfeeding is especially beneficial in areas where access to safe, clean food, and water is compromised (5). Nevertheless, many infants in low-income settings are introduced to non-breast milk foods early in life (6).

Breastfeeding is particularly important for HIV-infected infants, whose disease progresses more rapidly with replacement foods (6–10). One factor contributing to increased HIV disease progression in infants that are not exclusively breastfed may be exposure to foodborne, immunomodulatory toxins that could accelerate immune activation, a key predictor of HIV disease progression (11, 12). Ochratoxin A (OTA) is one of the most common small molecule mycotoxins worldwide, including in South Africa (13–15), and has been implicated in renal disease, immune modulation, and cancer (16). OTA is produced by a number of species of both *Aspergillus* and *Penicillium* fungi, which are found in soil (17–20) and water (21), but can also grow on food products (22, 23). Indeed, OTA contamination has been detected in a vast range of agricultural products, including cereals, grapes, spices, medicinal herbs, chocolate, and meat from animals raised on OTA-contaminated feed (24).

At high doses, OTA is cytotoxic, resulting in tumorigenesis, nephrotoxicity, and immunosuppression (25, 26). However, with lower levels of exposure, OTA induces excessive inflammation, including induction of TNF-α, IL-6, and IL-1 production by multiple cell types, both *in vitro* and in animal models (27–29). OTA’s immunomodulatory activity is believed to be mediated by its robust induction of reactive oxygen species, paired with suppression of oxidative stress response pathways, which in turn stimulate pro-inflammatory pathways (30, 31). This is particularly important in the context of HIV, since oxidative stress increases HIV replication, which may increase disease progression (32). Here, we provide evidence that OTA levels are elevated in non-breastfed South African infants and that this elevation is associated with T cell activation in HIV-exposed South African infants.

## Materials and Methods

### Study Participants

Infants evaluated in this study were enrolled at birth from the maternity ward at the Site B Clinic in Khayelitsha, Western Cape, South Africa, between 2010 and 2012. This study cohort has been previously described in Ref. (33). Inclusion criteria were term pregnancy (36 weeks gestational age or greater at birth), birth weight >2.4 kg, maternal age 18 years or older, known maternal HIV status, HIV DNA PCR negative at birth or at 6 weeks of age (where applicable), and no potential household tuberculosis contact. All mothers of eligible infants were verbally informed of potential risks and benefits of the study, and signed an IRB-approved consent form in their preferred language, either English or isiXhosa. At birth, 2, 6, 8, and 14 weeks of age, mothers completed an interviewer-assisted survey that included detailed information about the foods consumed by the infant, their health status since the last study visit, including receipt of vaccinations, and maternal health status, including the mother’s most recent CD4 count, if HIV-infected. In addition, infants received a full physical exam, including anthropometrics and evaluation for any oral or skin conditions. Finally, blood (EDTA by venipuncture; BD, CA, USA) was collected at each study visit. Two cohorts of infants were included, one cohort included infants born to HIV-infected mothers and were, therefore, HIV-exposed infants, and a second cohort of HIV-unexposed infants was also included where indicated. All HIV-infected mothers received antiretroviral treatment per South African national guidelines. Demographic data of these two cohorts are presented in Table 1. The low-income setting of this study resulted in difficulties with infant retention, and blood samples at some of the desired time points were not available. There were no demographic differences between infants that were retained in the study and those that were lost to follow-up (33). We acquired the largest number of samples at week 6, and a decreased number of samples at weeks 2 (28 infants), 8 (15 infants), and 14 (11 infants). The majority (>60%) of the infants at weeks 2, 8, and 14 were also evaluated at the week 6 time point.

**Table 1:** Demographics of study participants in each cohort.

	HIV-exposed (*n* = 58)	HIV-unexposed (*n* = 26)
Gender male, *n* (%)	20 (38.5)	N/A
Gestational age, weeks	40 (38–40)	40 (38–40)
Maternal CD4, cells/mm^3^	332 (238–477)	N/A
Birth weight, g	3,140 (2,820–3,300)	3,100 (2,940–3,240)
Breastfed, *n* (%)	15 (26%)	8 (31%)

*Values are reported as median vales and interquartile ranges or n and %, as indicated*.

*N/A, not available*.

Infants were defined as breastfed if they had consumed only breastmilk up to the time of sample collection, per maternal report. Non-breastfed infants were infants whose mother’s no longer fed them breast milk (i.e., replacement fed). Infants who consumed both breast milk and non-breast milk foods at the time of sample collection were considered mixed fed.

### Blood Processing

Blood samples were stored at room temperature in an air conditioned room (temperature no greater than 30°C) for no more than 6 h before transport. Blood was centrifuged, and plasma was aliquoted and frozen at −80°C. Peripheral blood mononuclear cells (PBMCs) were isolated from the remaining cells, using Ficoll (Sigma, MO, USA) density gradient separation. PBMCs were slowly cooled to −80°C in fetal bovine serum (FBS) (Biochrom, UK) + 10% DMSO (Sigma, MO, USA), and transferred to liquid nitrogen for storage.

### Assessment of T Cell Activation by Flow Cytometry

Frozen PBMCs were thawed at 37°C, washed, and resuspended in PBS (Sigma, MO, USA) + 1% FBS (Biochrom, UK), and cell counts were quantified with a Guava automated cell counter (EMD Millipore, Darmstadt, Germany).

Only infants who had not received BCG vaccination were included in these analyses, the influence of BCG vaccination on immune activation was assessed in a previous study (33). The focus on only non-BCG vaccinated infants enabled us to remove this potential immune stimulation as a confounder from the analysis, as BCG vaccination independently increases T cell activation (33, 34). Each sample was stained with optimized volumes of antibodies to evaluate T cell activation as previously published in Ref. (33). One million cells were stained in a 96-well plate with Live/Dead-Violet (Invitrogen, CA, USA) for 20 min at room temperature, then with anti-CCR5-APC (BD, CA, USA) at 37°C for 15 min, and then stained with anti-CD4-PerCP-Cy5.5 (BD, CA, USA), anti-CD38-PE-Cy7 (eBioscience, CA, USA), anti-HLA-DR-PE (BD, CA, USA), and anti-CD8-QDot605 (Invitrogen, CA, USA) for 20 min at room temperature. Samples were then washed with PBS, permeabilized with CytoFix/CytoPerm (BD, CA, USA), washed twice with Permawash (BD, CA, USA), and then stained intracellularly with anti-Ki-67-FITC and anti-CD3-AlexaFlour700 (BD, CA, USA) for 20 min at room temperature. A final wash was performed and then cells were resuspended in Cell Fix (BD, CA, USA), transferred to a polysterene FACS tube and collected on a Becton Dickinson LSR II flow cytometer within 24 h of staining. All flow cytometry data were compensated and analyzed, using Flow Jo v10 (Treestar, OR, USA). Samples with less than 80% lymphocyte viability were not included in the analysis (Live/Dead negative).

### Ochratoxin Quantification

Ochratoxin A was quantified in infant plasma by competitive ELISA in accordance with the manufacturer’s instructions (Helica, CA, USA). Each sample and control was processed in duplicate. Absorbance was measured on a SpectraMax plate reader (Molecular Devices, CA, USA) at 450 nm. OTA levels in each sample were calculated based on a 5-parameter regression of known standards. Values calculated below the lowest standard were assigned the value of the lowest standard. Interassay variability of the assay was minimal (CV 11.2%), based on repeat testing of 10 samples. Lower limit of detection was 80 pg/mL for HIV-exposed infants and 100 pg/mL for HIV-unexposed infants.

Formula and infant food samples were collected from the three non-breastmilk foods most commonly reported by mothers of infants in our study. All samples were advertised as “Baby Cereal,” procured from grocery stores in Cape Town and were manufactured in 2014. Sample 1 was Nestle’s Nestum Baby Cereal Regular and was composed of: wheat flour, brown sugar, flavoring, calcium carbonate, sodium phosphate, vitamins (A, B1, B2, B6, B12, C, D3, E, biotin, niacin, folic acid, pantothenic acid), maltodextrin, sodium chloride, ferrous fumarate, zinc sulfate, *Bifidobacterium lactis*, and potassium iodide. Sample 2 was Purity’s Baby’s First Cereal Rice and was composed of: rice flour, maltodextrin, non-hydrogenated vegetable fat, tricalcium phosphate, dicalcium phosphate, vitamins (A, C, D, pantothenic acid, thiamine, riboflavin, pyridoxine, biotin, folic acid, cyanocobalamin), and minerals (magnesium, iodine, iron zinc). Sample 3 was Nestle’s Cerelac Baby Cereal with Milk Regular and was composed of: wheat flour, skimmed milk powder, brown sugar, vegetable oil, flavoring, sodium chloride, calcium carbonate, sodium phosphate, maltodextrin, vitamins (A, B1, B2, B6, B12, C, D3, E, biotin, niacin, folic acid, pantothenic acid), ferrous fumarate, *B. lactis*, zinc sulfate, and potassium iodide. These samples were removed directly from their packaging and tested for OTA by LC-MS/MS, performed by Silliker Inc. Samples were suspended in acetonitrile, ascorbic acid, and water solution and extracted in a stomacher for 2 min. Samples were then cooled for 1 h at 2–8°C and an aliquot was diluted in water, and then vortexed and spun down. The diluted sample was then injected into LC–MS/MS [parent ion (M + H) 404, daughter ions 239 (quantitation trace) and 221 (confirmation trace)]. Lower limit of detection for this assay was 0.8 µg OTA per kilogram of food (LOD 0.8 ppb and LOQ 5 ppb).

### Measurement of Plasma Cytokines/Chemokines

Plasma samples from 0-, 2-, and 6-week time points from the infants who were not administered the BCG vaccine were thawed on ice. Prior to cytokine measurements, plasma was centrifuged at 10,000 *g* for 10 min to remove debris and supernatants were used to determine GM-CSF, IFN-α, IFN-γ, IL-1β, IL-8, CXCL-10 (IP-10), MCP-1, MIP-1β, TNF-α, IL-12p70, and IL-10 concentrations in plasma samples using MilliplexTM Human Cytokine kits (Millipore, MA, USA). The lower limit of detection of these assays range between 3.2 and 7.2 pg/mL for the cytokines measured. Data were collected using a Bio-Plex 200^TM^ Suspension Array Reader (Bio-Rad Laboratories, CA, USA) and analyzed using Bio-Plex manager software (version 4) (Bio-Rad Laboratories, CA, USA). Cytokine levels that were below the lower limit of detection of the assay were reported as the mid-point between the lowest concentrations measured for each cytokine and 0.

### Statistical Analysis

Statistics were performed in Prism 5 (Graphpad, CA, USA) and R (35). Unpaired *t*-tests were used after testing for normality with D’Agostino and Pearson omnibus normality test (*p*-value for all populations > 0.05). Where appropriate, multiple comparison correction was performed using the Holme stepdown approach (36). Spearman correlations were used for evaluating correlations. Multivariate analysis was performed in R, using the lm function and the following equation:

$$y=\text{OTA}+1.5^{\left(\text{gestational age}\right)}+\text{male}+{\text{log}}_{10}\left(\text{maternal cd4 count}\right)+{\text{log}}_{2}\left(\text{visit age}\right),$$

where *y* = immune activation marker of interest. If the parameter gained normal distribution with transformation, exponential or log transformation was applied to the parameter before further analysis.

## Results

### Cohort Demographics

To assess plasma levels of the mycotoxin OTA in HIV-exposed, uninfected infants, we evaluated samples from infants enrolled in two studies in Khayelitsha, South Africa (37), an informal settlement just outside of Cape Town, South Africa, which followed infants until 14 weeks of age. A subset analysis, based on availability of samples for these studies, was performed on 2-, 6-, 8-, and 14-week-old infants. There was a slight overrepresentation of female infants in the HIV-exposed infants (Table 1). In the HIV-exposed infants, maternal CD4 count ranged from 71 to over 1,000 cells/mm^3^, although the median CD4 count was above 200 cells/mm^3^. Consistent with the eligibility criteria, median gestational age was >38 weeks and birth weights ranged from 2,500 to 4,100 g.

### OTA Plasma Levels in South African Infants

**Figure 1:**
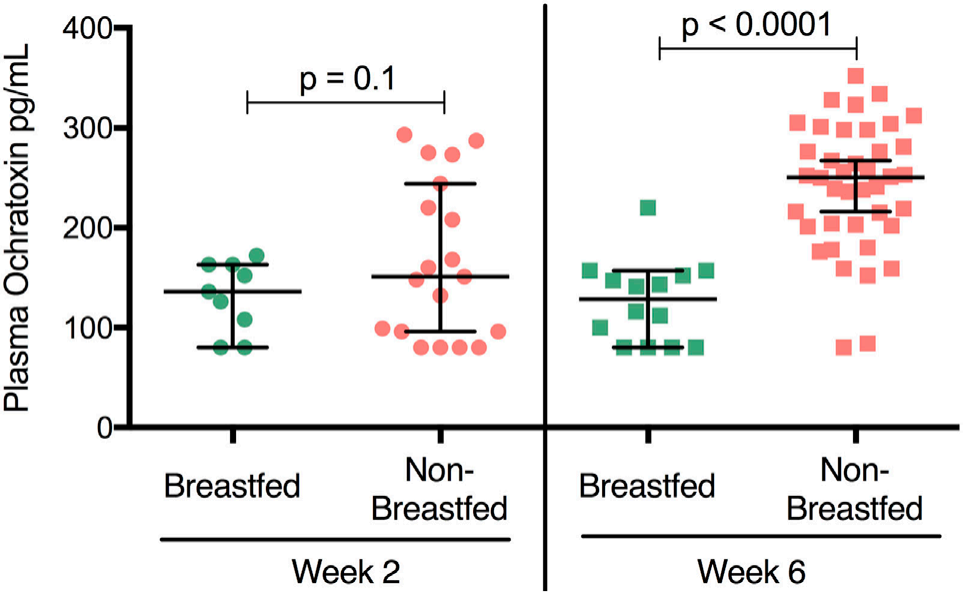
Plasma ochratoxin A (OTA) levels are elevated in non-breastfed, South African infants. 2-week-old breastfed (*n* = 9) and non-breastfed (*n* = 19) infants were comparable in their levels of plasma OTA (*p* = 0.1, Student’s *t*-test). In contrast, at 6 weeks of age non-breastfed (*n* = 42) infants showed higher levels of OTA (increased nearly two fold) compared to their breastfed counterparts (*n* = 14; *p* < 0.0001). Bars reflect the median OTA plasma level for each population. The lower limit of detection for the OTA ELISA assay was 80 pg/mL. All infants were HIV-exposed.

**Figure 2:**
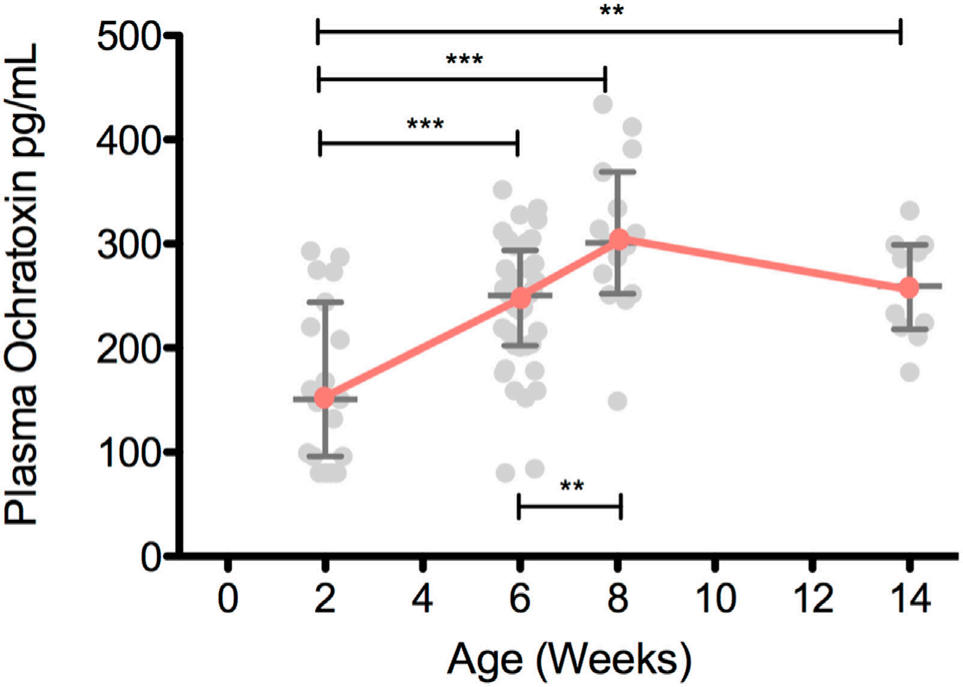
Plasma ochratoxin A (OTA) increases over the first 8 weeks of life in non-breastfed, South African infants. Plasma OTA levels in non-breastfed infants were assessed in 2-week-old (*n* = 19), 6-week-old (*n* = 40), 8-week-old (*n* = 15), and 14-week-old (*n* = 11) infants. Pairwise comparisons were tested with a Student’s *t*-test. All comparisons with a *p*-value less than 0.05 are shown above. Median and interquartile range are plotted for each time point. ***p < 0.001, **p < 0.01, *p < 0.05. All infants were HIV-exposed.

Ochratoxin A plasma levels at 2 weeks of age were relatively low for both breastfed and non-breastfed infants (131 and 151 pg/mL, respectively; Figure 1). However, at 6 weeks of age, OTA plasma levels increased in non-breastfed infants (151 to 250.5 pg/mL), while no increase was observed between 2 and 6 weeks in the exclusively breastfed infants (Figure 1). Longitudinal analysis of non-breast fed infants determined that OTA levels continued to increase in a linear fashion until 8 weeks of age, followed by a slight, but significant, decline at 14 weeks of age, the last time point available for analysis (Figure 2). The OTA levels detected in the plasma of South African infants are within the range of plasma levels observed in adults from OTA-endemic communities (16).

**Figure 3:**
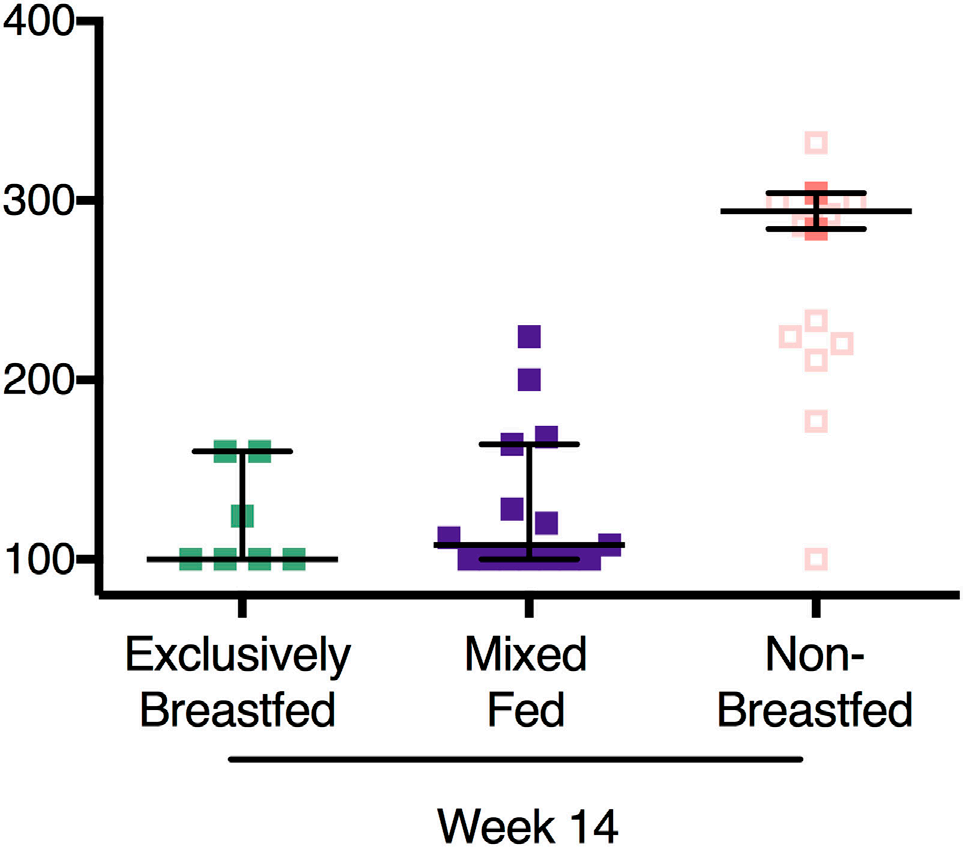
Recapitulation of ochratoxin A (OTA) exposure levels in HIV-exposed and -unexposed infants without elevation in OTA plasma levels in mixed fed infants. In filled colored squares: OTA plasma levels in HIV-unexposed infants that were exclusively breastfed, mixed fed, and non-breastfed infants at 14 weeks of age. In addition, plasma OTA levels from the non-breastfed, HIV-exposed infants at week 14 are also included (open squares). Bars reflect the median OTA plasma level for HIV-unexposed infants. *p*-Values reflect both HIV-exposed and HIV-unexposed infants.

In addition, OTA analysis was also performed on plasma from HIV-unexposed infants at 14 weeks of age. Since many HIV-negative mothers feed their infants both breastmilk and non-breastmilk foods (mixed feeding), we also included these infants in the comparison (Figure 3). Non-breastfed infant plasma contained elevated levels of plasma OTA when compared to both the exclusively breastfed and mixed fed infants (294 and 100 pg/mL, respectively). These levels were comparable to week 14 samples from the HIV-exposed infants (Figure 3, open red squares). Interestingly, mixed fed infants had low to intermediate levels of plasma OTA, suggesting that variable degrees of non-breastmilk food intake results in a broader range of plasma OTA. This finding provides evidence that elevated plasma OTA levels can be observed in both the HIV-exposed and HIV-unexposed infant groups.

### OTA Exposure and Immune Activation

**Figure 4:**
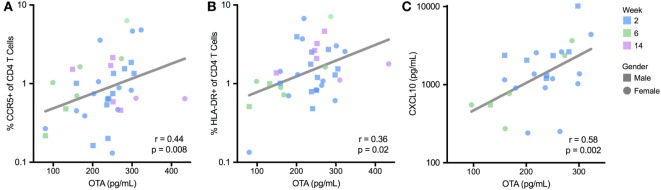
Plasma ochratoxin A (OTA) correlates with markers of immune activation in BCG-unvaccinated, non-breastfed infants. T cell activation, reported as a percentage of CCR5 **(A)** and HLADR-expressing CD4 T cells **(B)**, was quantified by flow cytometry, and plasma CXCL10 levels **(C)** were quantified by multiplex immunoassay. *R* and *p*-value reflect Spearman correlation. For **(A–C)**, lines reflect the best-fit, univariate, linear correlation. CXCL10 levels were not quantified for 8-week-old infants. All infants were HIV-exposed.

To evaluate the immunological effects associated with OTA exposure, the activation status of CD4 T cells was measured in infant PBMCs. Two T cell populations that were of particular interest were HIV target cells (those that co-express the HIV-receptor and co-receptor, CD4 and CCR5) (38) and the activated CD4 T cells (identified by MHC class II molecule HLA-DR expression) (39, 40). CCR5 and HLA-DR expression on CD4 T cells were evaluated at 2, 6, and 8 weeks of age in HIV-exposed, non-breastfed infants. In these infants, OTA plasma levels positively correlated with frequencies of CCR5 (*r* = 0.44; *p* = 0.008; Figure 4A) and HLA-DR-expressing CD4 T cells (*r* = 0.36; *p* = 0.02; Figure 4B). These findings indicate that increased levels of HIV target cells and CD4+ T cell activation, which promotes HIV replication, is associated with elevated levels of OTA (38, 41). In addition, a multi-analyte profiling panel was utilized to measure plasma levels of nine inflammatory markers. Of these, plasma levels of CXCL10/IP-10 significantly positively correlated with plasma OTA levels (*r* = 0.58; *p* = 0.002; Figure 4C). GM-CSF, IFN-α, IL12p70, and IL-10 were undetectable in most samples (LLD = 7.15, 3.2, 3.2, and 3.2 pg/mL, respectively). IFN-γ, IL-8, MCP-1, MIP-1β, TNF-α, and IL-10 levels did not correlate with OTA plasma levels (median = 6.4, 57.3, 556.4, 64.4, and 39.5 pg/mL, respectively). The relationship between OTA levels and CXCL10 persisted after adjusting for multiple comparisons.

To further evaluate these relationships, we performed a stepwise, multivariate regression, including variables that were selected *a priori*, based on their potential to influence immune activation; maternal HIV disease stage (measured by maternal CD4 count), infant age at sample collection, gender, and gestational age at birth, as well as OTA plasma levels. In this multivariate assessment, these three inflammatory markers (CCR5 and HLA-DR expression on CD4+ T cells and plasma CXCL10 levels) remained significantly associated with plasma levels of OTA (Table 2). Specifically, for every 100 pg/mL increase in plasma OTA levels, the percentage of CD4+ cells expressing CCR5 increased by 1.5-fold, the percentage of CD4+ cells expressing HLA-DR increased by 10-fold, and CXCL10 plasma levels increase by 2.5-fold. Indeed, in contrast to the other factors evaluated, only OTA plasma levels remained significantly correlated with these inflammatory markers.

**Table 2:** Ochratoxin A (OTA) is the only immune modulatory factor consistently correlated with immune activation in multivariate analysis.

	OTA (pg/mL)		Male gender		Gestational age		Maternal CD4		Visit age	
	Coefficient	*p*-Value	Coefficient	*p*-Value	Coefficient	*p*-Value	Coefficient	*p*-Value	Coefficient	*p*-Value
log_10_(CD4CCR5)	0.002	0.01	ns	ns	ns	ns	ns	ns	ns	ns
log_10_(CD4HLA-DR)	0.01	0.005	ns	ns	ns	ns	ns	ns	ns	ns
log_10_(CXCL10/IP-10)	0.004	0.004	0.24	0.10	ns	ns	ns	ns	ns	ns

*Coefficient and p-values for variables with p < 0.1 for the minimum, stepwise multivariate analyses*.

*NS: p > 0.1*.

### Sources of OTA in Exposure for South African Infants

The findings from this study suggest that South African infants in our study are exposed to OTA *via* a non-breast milk source. To assess potential sources of OTA in the diet of non-breastfed infants, we measured OTA levels in the four most common, non-breast milk foods given to infants in our study per maternal report. This included the infant formula provided free-of-charge to HIV-infected mothers, and three infant cereals commercially available in Khayelitsha, South Africa. Analysis of OTA levels determined that each of the four infant food samples had less than 0.8 µg OTA per kg of food, the lower limit of detection for the assay. Following this result, we hypothesize that alternative sources of OTA contamination exist.

## Discussion

Here, we evaluated an understudied immune-modulating factor, OTA, for its potential to influence immune activation in infants residing in Khayelitsha, South Africa. HIV is a major issue in this population as 33% of all pregnant women at this site tested positive for HIV in 2010 (58). The higher rate of disease progression in HIV-infected infants that are replacement fed has, historically, been attributed to poor nutritional intake or exposure to increased levels of pathogens (42–46). Our focus on non-breastfeeding infants at risk for acquiring HIV allows for a direct comparison between a patient population at risk of disease acquisition and an immune-modulating factor (OTA) that can influence target cells for viral infection. However, since we have no clinical outcomes reported in our study, we do not wish to overstate the role of OTA on HIV acquisition/disease progression or all-cause mortality.

Environmental and food contamination of OTA occur worldwide, but exposure levels vary significantly across the globe (47). Here we report, for the first time, that non-breast fed infants, but not exclusively breastfed or mixed fed infants, have elevated levels of OTA within the first weeks of life. In fact, the plasma levels of OTA detected in our study are consistent with the plasma levels observed in communities with sufficiently high levels of exposure that they are described as OTA-endemic (16). In addition, these increased levels of OTA correlated with elevated levels of activated CD4 T cells (CCR5+ and HLA-DR+) and an inflammatory chemokine associated with Th1 T cell mobilization and activation, CXCL10. Increased HLA-DR, CCR5, and CXCL10 expression has been implicated in the pathogenesis of key causes of infant mortality in Africa, including HIV (38, 48–50), lower-respiratory infections (51, 52), sepsis (53), encephalopathy (54), and meningitis (55). While we cannot rule out that plasma OTA levels are merely a biomarker for increased food antigen exposure, our study is the first to identify OTA at high levels in infants consuming non-breast milk foods. Therefore, OTA has the potential to increase morbidity and mortality in HIV-uninfected infants, and worsen HIV disease progression in HIV-infected infants.

It is important to consider our findings in the context of the nutritional reality faced by individuals in many low-income communities, including those living in Khayelitsha (56). It is well documented that deficiencies in antioxidants, such as vitamins C, E, and selenium, remain common in many countries, including South Africa (57). OTA impacts the immune system, in part, by inducing production of reactive oxygen species, while inhibiting cellular antioxidant pathways (30, 31). Therefore, deficits in exogenous antioxidants could potentiate OTA’s immune activation in communities with poor access to quality nutrition. This is particularly important in the context of HIV infection, since oxidative stress increases levels of immune activation and HIV viral replication (32). This mechanism suggests that addition of exogenous antioxidants may be an effective and feasible means of inhibiting OTA-mediated immune activation. This finding provides a potential mechanism for the increased morbidity and mortality of formula-fed infants in sub-Saharan Africa, regardless of HIV status.

Although the source of OTA has not been identified, the increase in OTA only in non-breastfed infants implicates non-breast milk food contamination, either during manufacturing or during storage or preparation in the home, as the source of this toxin. This source could, therefore, be from soil fungus in the home, or fungal contamination of water due to communal water sources and poor sanitation. We further speculate that since the formula and cereals tested were not determined to have detectable levels of OTA, storage and preparation techniques that minimize soil or water OTA contamination of formula and cereals would have beneficial health outcomes to infants, irrespective of HIV status. However, the greatest benefit will likely be realized in HIV-infected infants for whom elevated levels of oxidative stress and inflammation can exacerbate HIV disease progression.

Infants in South Africa are exposed to the food borne, soil-associated toxin OTA when consuming non-breast milk foods. Increased plasma OTA levels are associated with increased activation of immune cells with multiple functions, including HIV target cells. Identifying sources and reducing levels of OTA in infants from low-income countries would likely result in immune benefits, particularly in HIV-exposed or -infected infants.

## Ethics Statement

This study was carried out in accordance with the recommendations of University of Cape Town and University of Washington IRB Committees with written informed consent from all subjects. All subjects gave written informed consent in accordance with the Declaration of Helsinki. The protocol was approved by the University of Cape Town and University of Washington IRB Committees.

## Author Contributions

LW, DS, and HJ conceptualized this project. HJ oversaw the recruitment of study subjects. LW and HJ collected and analyzed the data. LW, MW, and BF created the tables and figures. LW, MW, BF, and DS prepared the manuscript. All authors provided leadership and advice on the design, implementation, and completion of the project.

## Conflict of Interest Statement

The authors declare that the research was conducted in the absence of any commercial or financial relationships that could be construed as a potential conflict of interest. The reviewer YZ and handling editor declared their shared affiliation.
